# Identification of Cleavage Sites Proteolytically Processed by NS2B-NS3 Protease in Polyprotein of Japanese Encephalitis Virus

**DOI:** 10.3390/pathogens10020102

**Published:** 2021-01-21

**Authors:** Abdul Wahaab, Ke Liu, Muddassar Hameed, Muhammad Naveed Anwar, Lei Kang, Chenxi Li, Xiaochun Ma, Abdul Wajid, Yi Yang, Umair Hassan Khan, Jianchao Wei, Beibei Li, Donghua Shao, Yafeng Qiu, Zhiyong Ma

**Affiliations:** Shanghai Veterinary Research Institute, Chinese Academy of Agricultural Sciences, Shanghai 200241, China; abdul.wahaab@uaf.edu.pk (A.W.); liuke@shvri.ac.cn (K.L.); mudasar386@gmail.com (M.H.); dr.naveed903@gmail.com (M.N.A.); kangshanglin1122@outlook.com (L.K.); lichenxihsy@outlook.com (C.L.); maxiaochun126@outlook.com (X.M.); abdulwajid44244@gmail.com (A.W.); yangyi997694874@outlook.com (Y.Y.); umair1143@gmail.com (U.H.K.); jianchaowei@shvri.ac.cn (J.W.); lbb@shvri.ac.cn (B.L.); shaodonghua@shvri.ac.cn (D.S.)

**Keywords:** Japanese encephalitis virus, NS2B-NS3 protease, cleavage site, polyprotein, substrate, pathogenesis

## Abstract

Understanding the proteolytic processing of polyprotein mediated by NS2B-NS3 protease contributes to the exploration of the mechanisms underlying infection of Japanese encephalitis virus (JEV), a zoonotic flavivirus. In this study, eukaryotic and prokaryotic cell models were employed to identify the cleavage sites mediated by viral NS2B-NS3 protease in JEV polyprotein. Artificial green fluorescent protein (GFP) substrates that contained the predicted cleavage site sequences of JEV polyprotein were expressed in swine testicle (ST) cells in the presence and absence of JEV infection, or co-expressed in *E. coli* with the recombinant NS2B-NS3 protease that was generated by fusing the N-terminal protease domain of NS3 to the central hydrophilic domain of NS2B. The cleavage of GFP substrates was examined by western blot. Among twelve artificial GFP substrates containing the cleavage site sequences predictively processed by host cell and/or NS2B-NS3 proteases, all sites were found to be cleaved by host cell proteases with different efficiencies. The sites at internal C, NS2A/NS2B, NS2B/NS3 and NS3/NS4A junctions, but not the sites at internal NS3, internal NS4A and NS4B/NS5 junctions were identified to be cleaved by JEV NS2B-NS3 protease. These data provide insight into the proteolytic processing of polyprotein, which is useful for understanding JEV replication and pathogenesis.

## 1. Introduction

Japanese encephalitis virus (JEV) is a zoonotic flavivirus belonging to the genus *Flavivirus* in the family *Flaviviridae* that comprises more than 70 species, such as dengue virus (DENV), West Nile virus (WNV), yellow fever virus (YFV) and Zika virus. It is endemic and the most substantial cause of human encephalitis in eastern and southern Asia with 30,000–50,000 cases annually [1,2]. In addition, JEV is responsible for reproductive disorders in pigs, with important impacts on both human public health and the pig industry [3]. JEV is a single-stranded, positive-sense RNA virus and is phylogenetically classified into five genotypes (genotype I to V) according to the nucleotide sequence of the E gene. Most isolates from JEV hosts belong to genotype I (GI) or genotype III (GIII) [4,5].

JEV genome is approximately 11 kb in length and encodes a single large polyprotein translated from an individual open reading frame. The polyprotein is approximately 3400 amino acids consisting of three structural proteins [capsid (C), pre-membrane/membrane (prM), envelope (E)] and seven non-structural proteins (NS1, NS2A, NS2B, NS3, NS4A, NS4B, and NS5) [6]. After translation, the polyprotein is proteolytically cleaved at junction sites between each viral protein by both host cell and/or viral proteases, which is essential for viral polyprotein maturation and viral replication. According to the knowledge of polyprotein processing of other flaviviruses, JEV protease is speculated to cleave the polyprotein at intergenic junctions of NS2A/NS2B, NS2B/NS3, NS3/NS4A and NS4B/NS5 as well as at the internal sites within C, NS3 and NS4A [7,8].

NS3 is JEV protease that is well conserved among JEV genotypes and contains two domains, an N-terminal protease domain and a C-terminal helicase domain coupled via a short flexible linker [9]. JEV NS2B is the cofactor of NS3 protease, correlating with stabilization and substrate recognition of NS3 protease [10]. The N-terminal protease domain (180 residues) of NS3 [NS3(pro)] interacts directly with a central 40-amino acid hydrophilic domain of NS2B (NS2B(H)) to form an active serine protease [10,11]. The active NS2B-NS3 protease is required for the proteolytic process of the polyprotein, and therefore plays an essential role in viral replication and pathogenesis. The NS2B-NS3 protease recognizes and cleaves a dibasic amino acid sequence motif that is highly conserved among flaviviruses and consists of two basic residues (K-R, R-R, R-K or occasionally Q-R) at the canonical P2 and P1 positions immediately preceding the cleavage site, followed by a small amino acid (G, S, or A) at the P1′ position [8].

The cleavage sites proteolytically processed by NS2B-NS3 protease in JEV polyprotein are extrapolated from the knowledge of other flaviviruses, such as YFV and DENV [8,11]. For example, NS2B-NS3 protease of YFV is responsible for cleaving the polyprotein at NS2A/NS2B, NS2B/NS3, NS3/NS4A, NS4A/NS4B and NS4B/NS5 junctions that contain the consensus sequence [G/A]RR↓[S/G] [12,13,14]. In fact, the cleavage sites proteolytically processed by NS2B-NS3 protease in JEV polyprotein have not been mapped. In this study, we identified the cleavage sites in JEV polyprotein using recombinant NS2B-NS3 protease and artificial green fluorescent protein (GFP) substrates containing the predicted cleavage site sequences in a prokaryotic cell model of *E. coli.*

## 2. Results

### 2.1. Alignment of Cleavage Site Sequences

**Figure 1:**
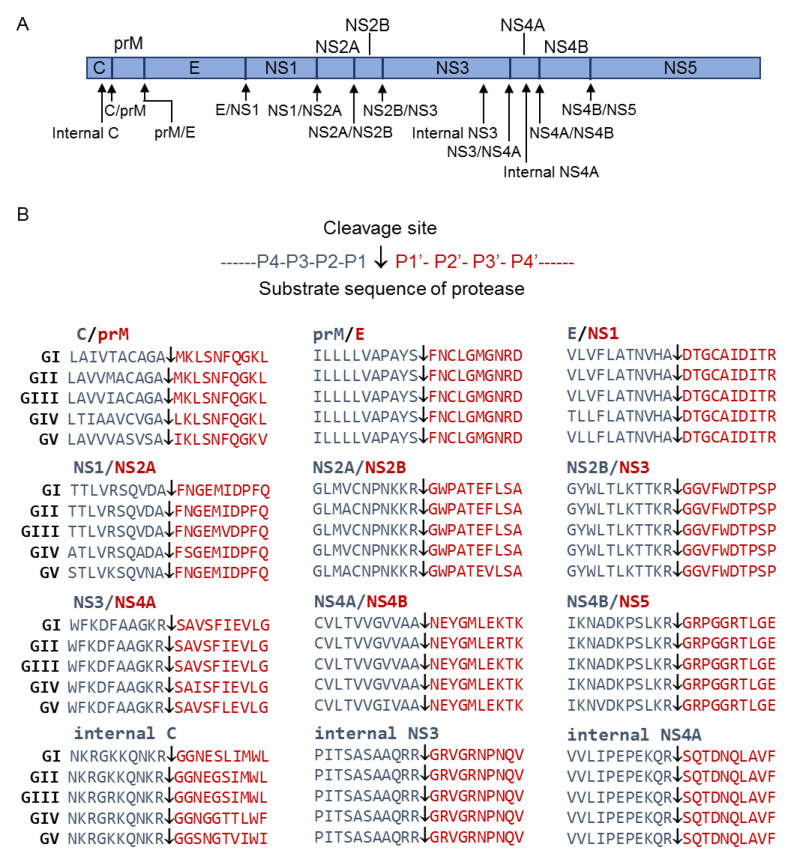
Cleavage sites predicted to be proteolytically processed by host cell and/or NS2B-NS3 proteases. (**A**) Schematic diagram of JEV polyprotein with the predicted cleavage sites. Arrowheads indicate the predicted cleavage sites. (**B**) Sequence alignment of the predicted cleavage sites for five genotypes of JEV. Arrowheads indicates the predicted cleavage sites.

JEV polyprotein after translation is cleaved by a combination of host cell and viral proteases to generate functional proteins. The cleavage is predicted to occur at the sites of internal C, C/prM, prM/E, E/NS1, NS1/NS2A, NS2A/NS2B, NS2B/NS3, internal NS3, NS3/NS4A, internal NS4A, NS4A/NS4B and NS4B/NS5 junctions (Figure 1A) [7,8]. Alignment of the predicted cleavage site sequences revealed that most of the sequences were identical or had high homology among the five genotypes of JEV, with exception of the sequences at the C/prM and NS1/NS2A junctions (Figure 1B). The sequence at the C/prM junction showed residue variations at P2, P3, P4 and P1′, while the sequence of NS1/NS2A junction displayed residue variation at P2, P3 and P2′.

### 2.2. Artificial GFP Substrates Are Cleaved by Host Cell Protease in Eukaryotic Cell Model

**Figure 2:**
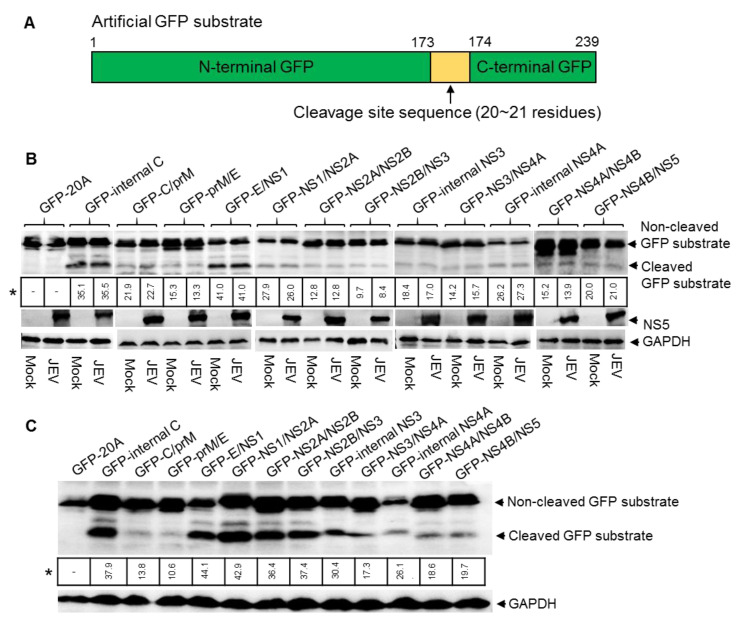
Detection of cleavage sites in eukaryotic cells. (**A**) Schematic diagram of the artificial GFP substrate. The sequence (20 ~ 21 residues) of cleavage site highlighted with yellow was inserted into GFP between residue 173 and 174. (**B**) ST cells were transfected with plasmids expressing the artificial GFP substrates and subsequently mock-infected or infected with JEV. Cleavage of GFP substrates in the transfectants was examined by western blot with antibodies specific to GFP. * indicates the percentage of cleaved GFP substrate/non-cleaved GFP substrate. (**C**) HEK293T cells were transfected with plasmids expressing the artificial GFP substrates and cleavage of GFP substrates in the transfectants was examined by western blot with antibodies specific to GFP.

JEV replicates in eukaryotic cells, such as swine testicular (ST), human embryonic kidney 293T (HEK293T) and baby hamster kidney (BHK) cell lines [15]; we therefore used a eukaryotic cell model to identify the sites cleaved by NS2B-NS3 protease in the JEV polyprotein. The artificial GFP substrates for JEV NS2B-NS3 protease were constructed by inserting the predicted cleavage site sequences of the JEV GIII strain (Figure 1B) into GFP at the position between residue 173 and 174 (Figure 2A). The resulting artificial GFP substrates containing the cleavage site sequences (GFP-internal C, GFP-C/prM, GFP-prM/E, GFP-E/NS1, GFP-NS1/NS2A, GFP-NS2A/NS2B, GFP-NS2B/NS3, GFP-internal NS3, GFP-NS3/NS4A, GFP-internal NS4A, GFP-NS4A/NS4B and GPF-NS4B/NS5) were used for analysis of the cleavage in the eukaryotic cells model. A control GFP substrate that contained a sequence of 20 alanine residues (GFP-20A) was used as a control.

ST cells were transfected with plasmids expressing the artificial GFP substrates in the presence and absence of JEV infection. The cleavage of artificial GFP substrates in the transfectants were examined by western blot with antibodies specific to GFP. No cleaved GFP band was observed in the cells expressing control GFP substrate (GFP-20A). However, a 21 kDa band corresponding to the N-terminal part of cleaved GFP substrate was observed in all cells expressing the artificial GFP substrates in the absence of JEV infection (Figure 2B), suggesting that the host cell protease was able to cleave all artificial GFP substrates. Comparison of the cleavage efficiencies of GFP substrates between the presence and absence of JEV infection showed no considerable difference (Figure 2B). We further selected several GFP substrates to compare the cleavage efficiencies between the presence and absence of JEV infection in BHK and HEK293T cells. Similar results were observed (Supplementary Figure S1A). These data suggested that host cell protease was able to cleave the proteolytic sites of JEV polyprotein. To confirm this observation, all artificial GFP substrates were further expressed in HEK293T and BHK cells in the absence of JEV infection and cleavage was examined by western blot. As expected, all GFP substrates were cleaved by host cell protease with different cleavage efficiencies (Figure 2C, Supplementary Figure S1B). The cleavage rates of several GFP substrates among ST, HEK293T and BHK cells were different. This apparent difference may be attributable to the differences in host protease activities among the cell types used. Noticeably, GFP-internal C, GFP-NS2A/NS2B, GFP-NS2B/NS3, GFP-internal NS3, GFP-NS3/NS4A, GFP-internal NS4A and GFP-NS4B/NS5 that are proposed to be cleaved by JEV NS2B-NS3 protease were also cleaved by host cell protease. Overall, these data suggested that host cell protease was able to cleave the proteolytic sites that are predicted to be cleaved by JEV NS2B-NS3 protease, thereby showing a difficulty in distinguishing the cleavage processed by host cell and NS2B-NS3 proteases in the eukaryotic cell model.

### 2.3. Identification of Cleavage Sites Proteolytically Processed by NS2B-NS3 Protease in E. Coli

**Figure 3:**
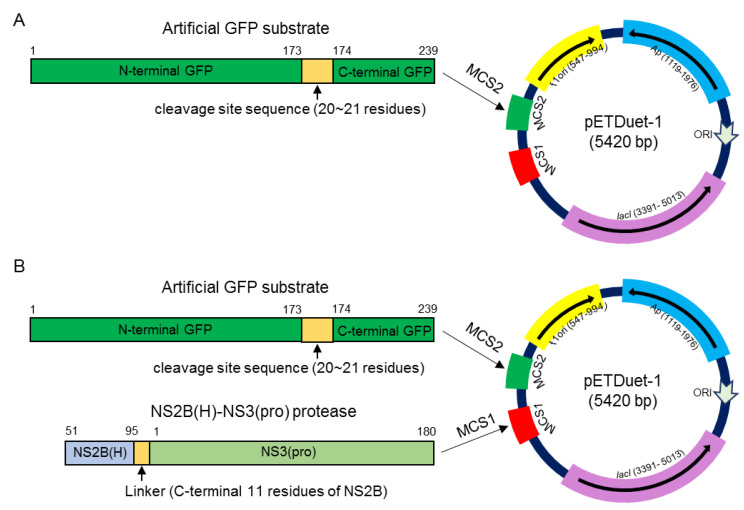
Schematic representation of recombinant plasmid. **(A)** Recombinant pETDuet-1 plasmid was engineered to express artificial GFP substrate at MCS2. **(B)** Recombinant pETDuet-1 plasmid was engineered to dually express NS2B(H)-NS3(pro) protease and artificial GFP substrate at MCS1 and MCS2, respectively.

Previous studies suggest that the recombinant NS2B-NS3 protease expressed in *E. coli* is likely to be folded correctly and shows proteolytic activity in *E. coli* [8,11]. Therefore, we selected the prokaryotic cell model of *E. coli* to identify the cleavage sites processed by JEV NS2B-NS3 protease. To this end, all artificial GFP substrates engineered in a prokaryotic expression plasmid pETDuet-1 (Figure 3A) were expressed in *E. coli* and the cleavage of artificial GFP substrates were examined by western blot to test whether the proteases of *E. coli* were able to cleave the GFP substrates. No cleaved GFP band was observed for all GFP substrates), suggesting that the proteases of *E. coli* were unable to cleave the sites engineered into the GFP substrates.

**Figure 4:**
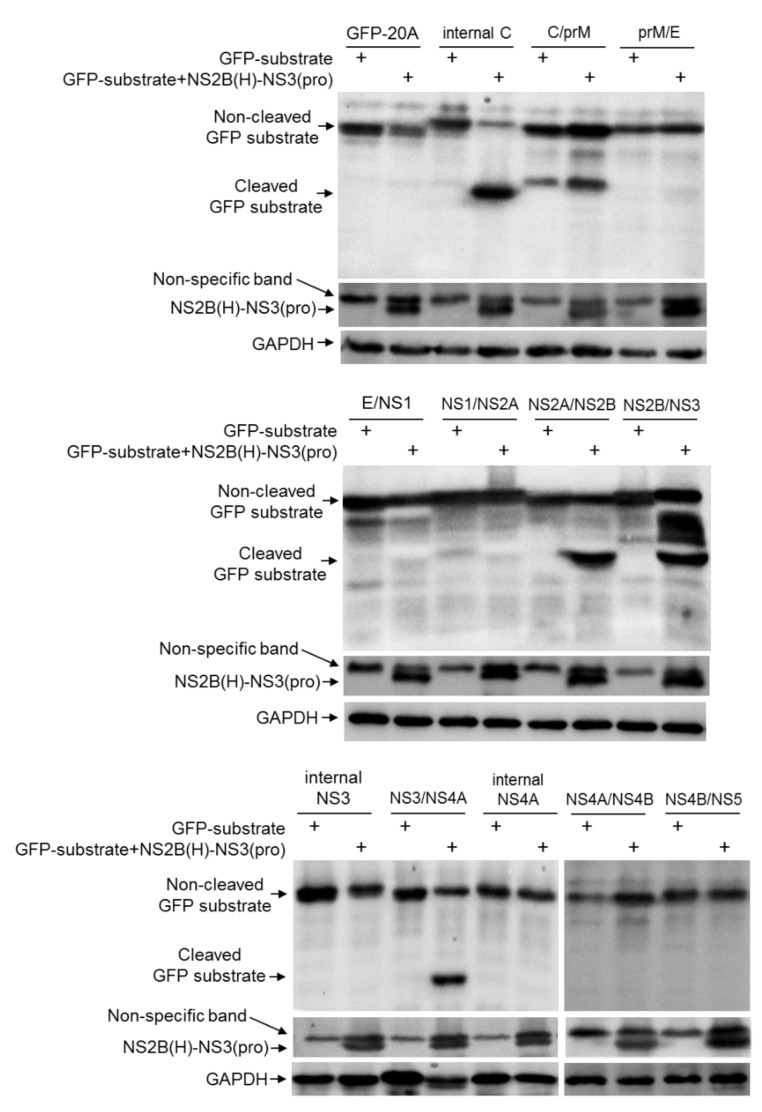
Detection of cleavage sites in *E. coli*. *E. coli* cells were transformed with recombinant plasmid dually expressing NS2B(H)-NS3(pro) protease and artificial GFP substrate or with recombinant plasmid expressing artificial GFP substrate alone. Cleavage of the artificial GFP substrate in *E. coli* was examined by western blot with antibodies specific to GFP. Expression of intact (non-cleaved) NS2B(H)-NS3(pro) protease was detected with antibodies specific to NS3.

JEV NS2B-NS3 protease activity is dependent on the association of NS3 with its cofactor NS2B(H). To produce active NS2B-NS3 protease, NS2B(H) was fused to NS3(pro) by a linker containing the C-terminal 11 amino acid residues of NS2B, as described previously [11], to generate the recombinant NS2B(H)-NS3(pro) protease of the JEV GIII strain (Figure 3B). The recombinant NS2B(H)-NS3(pro) protease and the artificial GFP substrates were co-expressed using the dual protein expression plasmid pETDuet-1 (Figure 3B) in *E. coli*. The cleavage of each GFP substrate was subsequently examined by western blot with antibodies specific to GFP. No cleaved GFP band was observed in the control *E. coli* expressing GFP substrate alone, confirming that the proteases of *E. coli* did not cleave the substrate sequences (Figure 4). However, a 21 kDa band corresponding to the N-terminal part of the cleaved GFP band was observed in *E. coli* co-expressing the recombinant NS2B(H)-NS3(pro) protease with GFP-internal C, GFP-NS2A/NS2B, GFP-NS2B/NS3 and GFP-NS3/NS4A, but not with GFP-C/prM, GFP-prM/E, GFP-E/NS1, GFP-NS1/NS2A, GFP-internal NS3, GFP-internal NS4A, GFP-NS4A/NS4B and GFP-NS4B/NS5. These results demonstrated that NS2B-NS3 protease was able to cleave the site at internal C, NS2A/NS2B, NS2B/NS3 and NS3/NS4A junctions.

### 2.4. Validation of the Cleavage Sites Proteolytically Processed by NS2B-NS3 Protease in E. Coli

**Figure 5:**
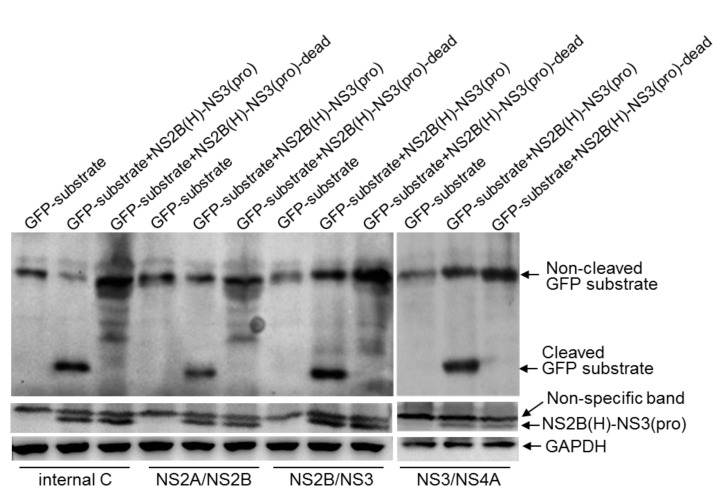
Validation of the identified cleavage sites in *E. coli*. *E. coli* cells were transformed with recombinant plasmid dually expressing active NS2B(H)-NS3(pro) protease and artificial GFP substrate (GFP-substrate+NS2B(H)-NS3(pro)), or inactive NS2B(H)-NS3(pro) protease and artificial GFP substrate (GFP-substrate+NS2B(H)-NS3(pro)-dead), or with recombinant plasmid expressing artificial GFP substrate alone. Cleavage of artificial GFP substrates in *E. coli* was examined by western blot with antibodies specific to GFP. Expression of intact (non-cleaved) NS2B(H)-NS3(pro) protease was detected with antibodies specific to NS3.

Substitution of a serine at position 135 located in the catalytic triad of NS3 with an alanine results in the inactivation of NS2B-NS3 protease [8]. To validate the cleavage sites identified with the recombinant NS2B(H)-NS3(pro) protease in *E. coli*, the recombinant NS2B(H)-NS3(pro) protease was inactivated by replacement of the catalytic serine at position 135 with an alanine to generate an inactive NS2B(H)-NS3(pro) protease [NS2B(H)-NS3(pro)-dead]. The recombinant NS2B(H)-NS3(pro)-dead protease was subsequently co-expressed with the GFP substrates in *E. coli* and the cleavage of GFP substrates was examined by western blot. In contrast to *E. coli* co-expressing the active recombinant NS2B(H)-NS3(pro) protease and the GFP substrates, in which a 21 kDa band of the cleaved GFP substrate was observed (Figure 5), no cleaved band was detected in *E. coli* co-expressing the recombinant NS2B(H)-NS3(pro)-dead protease with GFP-internal C, GFP-NS2A/NS2B, GFP-NS2B/NS3 and GFP-NS3/NS4A. These data confirmed that NS2B-NS3 protease was able to proteolytically cleave the sites at internal C, NS2A/NS2B, NS2B/NS3 and NS3/NS4A junctions.

## 3. Discussion

Understanding the cleavage sites mediated by NS2B-NS3 protease in the polyprotein is essential for exploring the mechanisms underlying the proteolytic processing of polyprotein as well as JEV replication. In this study, we used a prokaryotic cell model of *E. coli* to identify the cleavage sites in the polyprotein and observed that the sites at internal C, NS2A/NS2B, NS2B/NS3 and NS3/NS4A junctions were cleaved by NS2B-NS3 protease of JEV.

The proteolytic processing of JEV polyprotein is carried out by a combination of host cell- and virus-specified proteases to generate functional proteins that are essential for JEV replication and pathogenesis. According to the knowledge of polyprotein processing of other flaviviruses, JEV polyprotein processing is predicted as follows: the host cell protease mediates the cleavages at C/prM, prM/E, E/NS1, NS1/NS2A and NS4A/NS4B junctions, while NS2B-NS3 protease cleaves the polyprotein at intergenic junctions of NS2A/NS2B, NS2B/NS3, NS3/NS4A and NS4B/NS5 as well as at internal sites within C, NS3 and NS4A [7,8]. In this study, we tested whether JEV NS2B-NS3 protease was able to cleave the sites at C/prM, prM/E, E/NS1, NS1/NS2A and NS4A/NS4B junctions despite the fact that they are predicted to be cleaved by host cell protease, and observed no cleavage occurring in the presence of NS2B(H)-NS3(pro) protease, confirming that proteolytic processing of these proteins were mediated by host cell protease, not by NS2B-NS3 protease. Noticeably, among the cleavage sites predicted to be cleaved by NS2B-NS3 protease, the cleavage was observed at the sites of internal C, NS2A/NS2B, NS2B/NS3 and NS3/NS4A junctions, but not at internal NS3, internal NS4A and NS4B/NS5 junctions in the presence of NS2B(H)-NS3(pro) protease. These findings were analogous to the previous observations of other flaviviruses [8,9,11,12,13,16,17,18], in which the sites of internal C, NS2A/NS2B, NS2B/NS3 and NS3/NS4A are identified to be cleaved by flavivirus NS2B-NS3 proteases. However, the sites of internal NS3, internal NS4A and NS4B/NS5 that are processed by other flavivirus NS2B-NS3 protease were not cleaved by JEV NS2B-NS3 protease (Supplementary Table S1).

The functions of NS2B-NS3 proteases are well conserved among flaviviruses. However, multiple sequence alignment of flavivirus NS2B-NS3 proteases reveals that NS2B-NS3 protease of JEV GIII strain shares 76.3% (93.7% sequence similarity), 50.2% (79.5% sequence similarity) and 45.5% (76.9% sequence similarity) amino acid sequence identity with WNV, DENV2 and YFV, respectively. No conserved residues located at the binding pockets of NS2B-NS3 proteases are present among flaviviruses [11]. In addition to NS3 protease, the cofactor NS2B plays an essential role in stabilization and substrate recognition of NS3 protease [10]. JEV NS2B shows 67.2% amino acid sequence identity with WNV NS2B, and 18–34% identities with its orthologues from other flaviviruses. The central acid hydrophilic domain of NS2B is essential for activation of NS3 protease, and no conserved sequence in this domain is observed between JEV and other flaviviruses [10], despite the fact that the importance of this domain is similar among flaviviruses in terms of activation of NS3 protease [19,20]. The determinants for activation of JEV NS3 protease are possibly present at the positions of W62, G70, L75-D76-V77-K78-L79, G83 and E90-P91-G92-V93 in NS2B proteins [10], whereas the determinants in other flavivirus NS2B are identified at E52-L53-K54-K55 for YFV [20,21], W62, L75-S76-I77-T78-I79 and E89-E90-E91-E92 for DENV2 [22,23] and V89-E90-E91-T92 for DENV4 [19], respectively. These previous observations suggest possible differences in proteolytic activity and substrate recognition of NS2B-NS3 protease among flaviviruses, which might lead to a difference in the cleavage sites at the polyproteins. Indeed, the recombinant NS2B-NS3 proteases differ in their susceptibility to autolytic cleavage among JEV, WNV YFV and DENV [8]. Moreover, earlier studies demonstrate that a hybrid (DEN)NS2B-(JEV)NS3 protease, in which DENV NS2B is linked with JEV NS3, is able to cleave efficiently the JEV polyprotein, whereas a hybrid (JEV)NS2B-(DEN)NS3 protease, in which JEV NS2B is fused to DENV NS3, is inactive [10,24]. These previous observations suggest the differences in the conformational space between NS2B-NS3 proteases of different flaviviruses, and therefore might lead to the differences in substrate recognition and affinity. Overall, these previous observations might explain the difference in cleavage sites mediated by NS2B-NS3 protease in the polyprotein between JEV and other flaviviruses.

JEV replicates in susceptible eukaryotic cells, such as ST, BHK and HEK293T cells [15], which were supposed to be suitable as in vitro models for mapping the cleavage sites mediated by NS2B-NS3 protease in JEV polyprotein. However, we observed that host cell protease cleaved the sites predicted to be cleaved by NS2B-NS3 protease in ST, BHK and HEK293T cells. We further compared the cleavage efficiencies of artificial GFP substrates between mock- and JEV-infected groups, because we speculated that the cleavage rates of GFP substrates in JEV-infected groups might be higher than those in mock-infected groups. However, no considerable differences in the cleavage efficiencies of artificial GFP substrates were observed between mock- and JEV-infected groups. This result may be attributable to the fact that the artificial GFP substrates may be unable to translocate into the specific subcellular locations where viral NS2B-NS3 protease is present to recognize and cleave JEV polyprotein [8]. Overall, these data suggest that the susceptible eukaryotic cells were not suitable for mapping the cleavage site mediated by NS2B-NS3 protease. We therefore used the prokaryotic cell model of *E. coli* to identify the cleavage sites mediated by NS2B-NS3 protease. The prokaryotic cell model of *E. coli* has been used to analyze the proteolytic activity of flavivirus NS2B-NS3 proteases [8,23,25,26]. However, the proteolytic activity of recombinant NS2B-NS3 protease expressed in *E. coli* might not fully represent its proteolytic activity in flavivirus-infected eukaryotic cells, because some cofactors from host cells and/or viral proteins play a role in assistance of the proteolytic processing of polyprotein mediated by NS2B-NS3 protease. For example, DNAJC14, a heat shock protein 40 cochaperone, affects the cleavage of YFV NS3/NS4A and NS4A/NS2K and alters NS3-to-NS3-4A ratios, suggesting that chaperone-assisted protein folding is critical for YFV NS3/NS4A cleavage [27]. In addition to the prokaryotic cell model of *E. coli*, we used the truncated NS2B and NS3 to form an active recombinant NS2B(H)-NS3(pro) protease, which might not fully represent the proteolytic activity of wild type NS2B-NS3 protease. Therefore, we cannot exclude a possibility that NS2B-NS3 protease is able to cleave additional sites in the polyprotein in JEV-infected eukaryotic cells, besides the cleavage sites (internal C, NS2A/NS2B, NS2B/NS3 and NS3/NS4A) identified in this study. Furthermore, the NS2B(H)-NS3(pro) protease of GIII strain used in this study shared approximately 90% amino acid sequence identity with other JEV genotypes (GI, GII, GIV and GV). We cannot completely exclude the possibility that this difference in amino acid sequence of NS2B-NS3 protease between GIII and other genotypes may result in a different specificity in recognition and cleavage of substrates. Future experiments are needed to clarify whether NS2B-NS3 protease proteolytically processes the cleavage at internal NS3, internal NS4A and NS4B/NS5 junctions that are predicted to be cleaved by NS2B-NS3 protease as well as other unknown sites.

In conclusion, host cell protease was able to cleave all predicted sites at internal C, C/prM, prM/E, E/NS1, NS1/NS2A, NS2A/NS2B, NS2B/NS3, internal NS3, NS3/NS4A, internal NS4A, NS4A/NS4B and NS4B/NS5 junctions in JEV polyprotein. Among the sites predicted to be cleaved by NS2B-NS3 protease, the sites at internal C, NS2A/NS2B, NS2B/NS3 and NS3/NS4A junctions, but not at internal NS3, internal NS4A and NS4B/NS5 junctions in JEV polyprotein were identified to be cleaved by NS2B-NS3 protease in the prokaryotic cell model of *E. coli*. These data provide an insight into the proteolytic processing of polyprotein that would be useful for understanding JEV replication and pathogenesis, and could also facilitate development of rational drug design using viral proteases as a therapeutic drug target.

## 4. Materials and Methods

### 4.1. Virus and Cells

JEV GIII strain SH15 (GenBank no. MH753130.1) used for viral infection and cloning of the cleavage site and NS2B-NS3 protease sequences was isolated from mosquito in 2016 [28]. Swine testicular (ST), human embryonic kidney 293T (HEK293T) and baby hamster kidney (BHK) cell lines were cultured in Dulbecco’s modified Eagle’s medium (DMEM) containing 10% fetal bovine serum (FBS) (Gibco, Thermo Fisher Scientific, Waltham, MA, USA), 100 μg/mL streptomycin and 100 IU/mL penicillin. JEV infection was performed as described previously [29].

### 4.2. Sequence Alignment

Amino acid sequences of five genotypes of JEV including GI SH7 strain (GenBank no. MH753129.1), GII FU strain (GenBank no. AF217620.1), GIII SH15 strain (GenBank no. MH753130.1), GIV JKT6468 strain (GenBank no. AY184212.1) and GV XZ0934 strain (GenBank no. JF915894.1) were downloaded from GenBank. The sequences of the predicted cleavage sites at internal C, C/prM, prM/E, E/NS1, NS1/NS2A, NS2A/NS2B, NS2B/NS3, internal NS3, NS3/NS4A, internal NS4A, NS4A/NS4B and NS4B/NS5 junctions for five genotypes were aligned using SnapGene (GSL Biotech LLC, San Diego, CA, USA) and MegAlign (DNASTAR Inc, Madison, WI, USA) softwares.

### 4.3. Generation of Artificial GFP Substrate

Sequences encoding the predicted cleavage sites at internal C, C/prM, prM/E, E/NS1, NS1/NS2A, NS2A/NS2B, NS2B/NS3, internal NS3, NS3/NS4A, internal NS4A, NS4A/NS4B and NS4B/NS5 junctions (Figure 1) were cloned from JEV GIII SH15 strain by reverse transcription-polymerase chain reaction (RT-PCR) [30].with respective primers and inserted into pEGFP-C1 plasmid (BD Biosciences Clontech, Mountain View, CA, USA) by overlap extension PCR using special designed oligo dT primer pairs (Shanghai Sunny Biotech, Shanghai, China) to generate recombinant GFP plasmids expressing artificial GFP substrate. The resulting artificial GFP substrates consisted of the N-terminal part (amino acid 1 to 173) and the C-terminal part (amino acid 174 to 239) of GFP linked by the predicted cleavage site sequences. A sequence of 20 alanine residues was inserted between the N-terminal and C-terminal parts of GFP to generate a control GFP substrate. All primers used are listed in Supplementary Table S2.

### 4.4. Construction of Plasmid Dually Expressing NS2B(H)-NS3(Pro) Protease and Artificial GFP Substrate

Total RNAs were extracted from JEV GIII SH15 strain infected BHK cells using TRIzol reagent (Thermo Fisher Scientific, Waltham, MA, USA) according to the manufacturer’s directions. Reverse transcription was performed to generate cDNA using PrimeScript RT reagent kit with gDNA Eraser (TaKaRa, Kyoto, Japan). Sequence encoding the C-terminal part (amino acid 51 to 131) of NS2B and the N-terminal part (amino acid 1 to 180) of NS3 was amplified and inserted into the multi-cloning site 1 (MCS1) of dual protein expression plasmid pETDuet-1 (Novagen, Beijing, China) through EcoRI and NotI restriction sites. The sequence encoding the fragment from residue 96 to 120 of NS2B was deleted from the recombinant plasmid pETDuet-1 by PCR based site directed mutagenesis [31] using Pfu ultra II Fusion HS DNA polymerase (Agilent, Santa Clara, CA, USA) to generate a recombinant plasmid expressing the active recombinant NS2B(H)-NS3(pro) protease [11]. The sequence encoding the artificial GFP substrate was enzymically excised from the recombinant GFP plasmid and inserted into the multi-cloning site 2 (MCS2), site of the recombinant plasmid pETDuet-1 expressing the active recombinant NS2B(H)-NS3(pro) protease. To inactivate the activity of recombinant NS2B(H)-NS3(pro) protease [8], a serine at residue 135 was replaced with an alanine by PCR based site directed mutagenesis [31]. To express the artificial GFP substrate alone, the sequence encoding the artificial GFP substrate was enzymically excised from the recombinant GFP plasmid and inserted into the MCS2 site of the plasmid pETDuet-1. All primers used are listed in Supplementary Table S2.

### 4.5. Detection of Cleavage Sites in Eukaryotic Cells

ST, BHK and HEK293T cells were transfected with the recombinant GFP plasmid expressing the artificial GFP substrate using Lippofectamine 3000 (Invitrogen, Carlsbad, CA, USA) according to the manufacturer’s protocol and incubated at 37 °C for 12 h. The transfectants were subsequently mock-infected or infected with JEV SH15 strain at a multiplicity of infection (MOI) of 5 and harvested 36 h post-infection. Cleavage of the artificial GPF substrates was detected by western blot with antibodies specific to GFP (GFP (D5.1) XP Rabbit mAb, Cell Signaling Technology, Danvers, MA, USA), as described previously [32]. The viral NS5 and glyceraldehyde-3-phosphate dehydrogenase (GAPDH) were examined by anti-NS5 polyclonal antibodies (GeneTex, Irvine, CA, USA) and anti-GAPDH monoclonal antibody (Proteintech, Chicago, IL, USA), respectively.

### 4.6. Detection of Cleavage Sites in E. Coli

*E. coli* BL-21 (DE3) cells were transformed with the recombinant plasmid pETDuet-1 expressing NS2B(H)-NS3(pro) protease and artificial GFP substrate and incubated at 37 °C until OD600 reached 0.6. Expression was induced by isopropyl b-D-1-thiogalactopyranoside at a final concentration of 1.5 mM. After 24 h incubation at 37 °C, the cells were harvested by centrifugation at 12,000 × *g* for 5 min at 4 °C and the pellets were resuspended in phosphate buffered saline (PBS) and re-centrifugated at 12,000 × *g* for 5 min at 4 °C. The resulting pellets were subjected to western blot analysis with respective antibodies, as described previously [32]. Cleavage of the artificial GFP substrate was probed using anti-GFP antibodies (GFP (D5.1) XP Rabbit mAb). The expression of NS2B(H)-NS3(pro) protease was measured by antibodies specific to NS3 [33].

## Data Availability

Not applicable.

## Supplementary Materials

The following are available online at https://www.mdpi.com/2076-0817/10/2/102/s1. Figure S1. Detection of cleavage sites in eukaryotic cells. (A) BHK and HEK293T cells were transfected with plasmids expressing the artificial GFP substrates and subsequently mock-infected or infected with JEV. Cleavage of GFP substrates in the transfectants was examined by western blot with antibodies specific to GFP. (B) BHK cells were transfected with plasmids expressing the artificial GFP substrates and cleavage of GFP substrates in the transfectants was examined by western blot with antibodies specific to GFP. * indicates the percentage of cleaved GFP substrate/non-cleaved GFP substrate. Table S1. Cleavage sites processed by flavivirus NS2B/NS3 proteases. Table S2. Primers sequences used in this study.
