# Supplementary material for: Identification of Cleavage Sites Proteolytically Processed by NS2B-NS3 Protease in Polyprotein of Japanese Encephalitis Virus
Source: Pathogens. 2021 Jan 21;10(2):102. doi: 10.3390/pathogens10020102 (PMC7911949; doi:10.3390/pathogens10020102)
Supplement: Supplementary file 1 [file pathogens-10-00102-s001.zip › Supplementary Table 2 primers used(1).docx]

**Supplementary Table S2.** Primers sequences used in this study.

| **Primer name** | **Sequence (5́-3́)** | **Purpose** | **Restriction site** |
| --- | --- | --- | --- |
| **GFP recombinant** | **GAATTC**ATGGTGAGCAAGGGCGAGG | To amplify GFP | EcoRI |
|  | **GCGGCCGC**TTACTTGTACAGCTCGTCCA |  | NotI |
| **GFP-**  **internal C** | CCTCCTCTTTTGTTTTGCTTTCTGCCCCGCTTGTTCACGGCCTCGATGTTGTG | To generate internal C substrate |  |
|  | AGCAAAACAAAAGAGGAGGAAATGAAGGCTCAATCATGTGGCTCGACGGCGGCGTGCA |  |  |
| **GFP-**  **C/prM** | TTCATTGCTCCTGCGTAAGCTATGACAACTGCCAACTCGATGTTGTGGC | To generate C/prM substrate |  |
|  | CAGGAGCAATGAAGTTGTCGAATTTCCAGGGGAAGCTTGACGGCGGCGTGCAGC |  |  |
| **GFP-**  **prM/E** | CAATTAAAACTGTAAGCCGGAGCGACCAGCAGCAGGAGGATCTCGATGTTGTGGCG | To generate prM/E  substrate |  |
|  | GGCTTACAGTTTTAATTGTCTGGGAATGGGCAATCGTGACTTCGACGGCGGCGTGCAGC |  |  |
| **GFP-**  **E/NS1** | CAGTGTCAGCATGCACATTGGTCGCTAAGAACACGAGCACCTCGATGTTGTGGCG | To generate  E/NS1 substrate |  |
|  | GTGCATGCTGACACTGGATGTGCCATTGACATCACAAGAAAAGAGGACGGCGGCGTGCAGC |  |  |
| **GFP-**  **NS1/NS2A** | CCATTGAAAGCATCAACCTGCGATCTGACGAGTGTTGTCTCGATGTTGTGGCG | To generate  NS1/NS2A substrate |  |
|  | GTTGATGCTTTCAATGGTGAAATGGTTGACCCTTTTCAGCTGGACGGCGGCGTGCAGC |  |  |
| **GFP-**  **NS2A/NS2B** | CACCCCCTCTTCTTGTTTGGGTTGCAGACCATTAGTCCGGCCTCGATGTTGTGGCG | To generate  NS2A/NS2B substrate |  |
|  | CAAGAAGAGGGGGTGGCCAGCCACCGAGTTCCTTTCAGCGGACGGCGGCGTGCAGC |  |  |
| **GFP-**  **NS2B/NS3** | AACACGCCCCCTCTTTTTGTTGTTTTTAAAGTGAGCCAATAACCCTCGATGTTGTGGCG | To generate  NS2B/NS3 substrate |  |
|  | CAAAAAGAGGGGGCGTGTTTTGGGACACGCCATCCCCAAAACCTGACGGCGGCGTGCAG |  |  |
| **GFP-**  **internal NS3** | ACTCTACCTCTCCGTTGGGCTGCACTTGCACTGGTTATGGGGGACTCGATGTTGTGGCG | To generate  internal NS3 substrate |  |
|  | CGGAGAGGTAGAGTGGGCAGGAATCCTAACCAAGTTGACGGCGGCGTGCAGC |  |  |
| **GFP-**  **NS3/NS4A** | GGCTGATCTCTTGCCCGCTGCAAAATCCTTAAACCACTTGAGCTCGATGTTGTGGCG | To generate  NS3/NS4A substrate |  |
|  | CGGGCAAGAGATCAGCCGTCAGTTTCATAGAGGTGCTCGGTGACGGCGGCGTGCAGC |  |  |
| **GFP-**  **internal NS4A** | GTCTGTGACCTCTGTTTTTCTGGTTCCGGGATGAGGACCACCATCTCGATGTTGTGGCG | To generate  Internal NS4A substrate |  |
|  | AACAGAGGTCACAGACAGACAACCAGTTGGCAGTGTTTGACGGCGGCGTGCAGC |  |  |
| **GFP-**  **NS4A/NS4B** | CTCGTTTGCTGCCACCACTCCAACCACGGTCAAGACGCACTCGATGTTGTGGCG | To generate  NS4A/NS4B substrate |  |
|  | TGGCAGCAAACGAGTACGGGATGCTAGAAAAAACCAAAGACGGCGGCGTGCAGC |  |  |
| **GFP-**  **NS4B/NS5** | CCTTCCTTTCAAGGAGGGCTTGTCAGCGTTCTTGATGAGCTCGATGTTGTGGCG | To generate  NS4B/NS5 substrate |  |
|  | CTTGAAAGGAAGGCCCGGGGGCAGGACGCTAGGGGAGGACGGCGGCGTGCAGC |  |  |
| **NS2B-NS3-**  **pETDuet-1** | **GAATTC**GATGGTGTCAGGAAAAGCAACA | To amplify  NS2B-NS3 protease for MCS1 | EcoRI |
|  | **GCGGCCGC**TTATCTCTTTCTCAACATGTT |  | NotI |
| **NS2B-NS3pro mutagenesis** | TTGATGATCCCGGTGTTCCAGGTTATTGGCTCACTTTAAA | To delete a part of NS2B residues |  |
|  | TTTAAAGTGAGCCAATAACCTGGAACACCGGGATCATCAA |  |  |
| **NS2B-NS3pro inactive-mutagenesis** | ATTACCCGCGAGGAACAGCAGGCTCACCCATTCTGGATTC | To inactivate NS2B-NS3 protease |  |
|  | GAATCCAGAATGGGTGAGCCTGCTGTTCCTCGCGGGTAAT |  |  |
| **GFP-**  **pETDuet-1** | **CATATG**ATGGTGAGCAAGGGCGAGGAGCTG | To amplify GFP for pETDuet-1  MCS2 | NdeI |
|  | **GATATC**CACTTGTACAGCTCGTCCAT |  | EcoRV |

Bold and underlined sequences are inserted restriction sites.
