# Supplementary material for: Identification of Cleavage Sites Proteolytically Processed by NS2B-NS3 Protease in Polyprotein of Japanese Encephalitis Virus
Source: Pathogens. 2021 Jan 21;10(2):102. doi: 10.3390/pathogens10020102 (PMC7911949; doi:10.3390/pathogens10020102)
Supplement: Supplementary file 1 [file pathogens-10-00102-s001.zip › pathogens-1029840-supplementary-fig-1-final(1).docx]

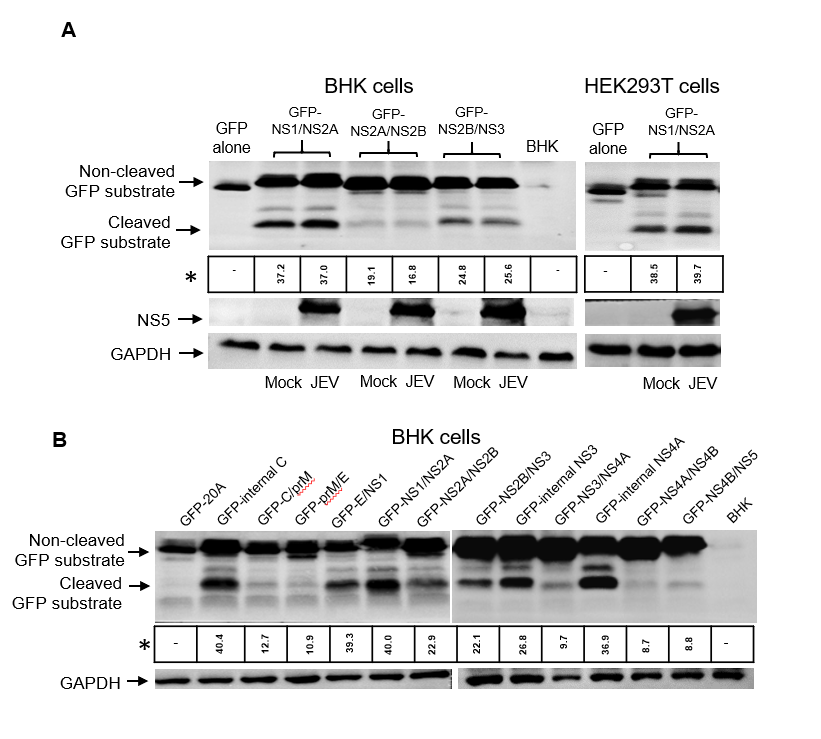


**Supplementary Fig. 1:** Detection of cleavage sites in eukaryotic cells. **(A)** BHK and HEK293T cells were transfected with plasmids expressing the artificial GFP substrates and subsequently mock-infected or infected with JEV. Cleavage of GFP substrates in the transfectants was examined by western blot with antibodies specific to GFP. **(B)** BHK cells were transfected with plasmids expressing the artificial GFP substrates and cleavage of GFP substrates in the transfectants was examined by western blot with antibodies specific to GFP. * indicates the percentage of cleaved GFP substrate/non-cleaved GFP substrate.
