# Supplementary material for: Identification of Cleavage Sites Proteolytically Processed by NS2B-NS3 Protease in Polyprotein of Japanese Encephalitis Virus
Source: Pathogens. 2021 Jan 21;10(2):102. doi: 10.3390/pathogens10020102 (PMC7911949; doi:10.3390/pathogens10020102)
Supplement: Supplementary file 1 [file pathogens-10-00102-s001.zip › Supplementary Table 1 Cleavage sites processed by flavivirus NS2B-NS3 proteases(1).docx]

**Supplementary Table S1 Cleavage sites processed by flavivirus NS2B-NS3 proteases**

| Flavivirus | Internal C | NS2A/NS2B | NS2B/NS3 | Internal NS3 | NS3/NS4A | Internal NS4A | NS4B/NS5 | Reference |
| --- | --- | --- | --- | --- | --- | --- | --- | --- |
| JEV | √ | √ | √ | × | √ | × | × | This study |
| WNV | √ | √ | √ | √ | √ | √ | √ | [1] [2-4] |
| YFV | - | √ | √ | - | √ | √ | √ | [5-7] |
| DENV | - | √ | √ | - | √ | - | √ | [8, 9] |

√, site identified by experiments. ×, site not cleaved by JEV NS2B-NS3 protease. -, no data found.

**References**

1. Bera, A. K.; Kuhn, R. J.; Smith, J. L., Functional characterization of cis and trans activity of the Flavivirus NS2B-NS3 protease. *The Journal of biological chemistry* **2007,** 282, (17), 12883-92.

2. Nall, T. A.; Chappell, K. J.; Stoermer, M. J.; Fang, N. X.; Tyndall, J. D.; Young, P. R.; Fairlie, D. P., Enzymatic characterization and homology model of a catalytically active recombinant West Nile virus NS3 protease. *The Journal of biological chemistry* **2004,** 279, (47), 48535-42.

3. Chappell, K. J.; Stoermer, M. J.; Fairlie, D. P.; Young, P. R., Insights to substrate binding and processing by West Nile Virus NS3 protease through combined modeling, protease mutagenesis, and kinetic studies. *The Journal of biological chemistry* **2006,** 281, (50), 38448-58.

4. VanBlargan, L. A.; Davis, K. A.; Dowd, K. A.; Akey, D. L.; Smith, J. L.; Pierson, T. C., Context-Dependent Cleavage of the Capsid Protein by the West Nile Virus Protease Modulates the Efficiency of Virus Assembly. *Journal of virology* **2015,** 89, (16), 8632-8642.

5. Preugschat, F.; Lenches, E. M.; Strauss, J. H., Flavivirus enzyme-substrate interactions studied with chimeric proteinases: identification of an intragenic locus important for substrate recognition. *Journal of virology* **1991,** 65, (9), 4749-58.

6. Chambers, T. J.; Grakoui, A.; Rice, C. M., Processing of the yellow fever virus nonstructural polyprotein: a catalytically active NS3 proteinase domain and NS2B are required for cleavages at dibasic sites. *Journal of virology* **1991,** 65, (11), 6042-50.

7. Lin, C.; Amberg, S. M.; Chambers, T. J.; Rice, C. M., Cleavage at a novel site in the NS4A region by the yellow fever virus NS2B-3 proteinase is a prerequisite for processing at the downstream 4A/4B signalase site. *Journal of virology* **1993,** 67, (4), 2327-35.

8. Falgout, B.; Pethel, M.; Zhang, Y. M.; Lai, C. J., Both nonstructural proteins NS2B and NS3 are required for the proteolytic processing of dengue virus nonstructural proteins. *Journal of virology* **1991,** 65, (5), 2467-75.

9. Leung, D.; Schroder, K.; White, H.; Fang, N. X.; Stoermer, M. J.; Abbenante, G.; Martin, J. L.; Young, P. R.; Fairlie, D. P., Activity of recombinant dengue 2 virus NS3 protease in the presence of a truncated NS2B co-factor, small peptide substrates, and inhibitors. *The Journal of biological chemistry* **2001,** 276, (49), 45762-71.
